# Differential impact of quarantine policies for recovered COVID-19 cases in England: a case cohort study of surveillance data, June to December 2020

**DOI:** 10.1186/s12889-022-14254-x

**Published:** 2022-10-14

**Authors:** Rachel Merrick, Dimple Chudasama, Joe Flannagan, Ines Campos-Matos, Annabelle Howard, Renu Bindra, O Noël Gill, Gavin Dabrera, Theresa Lamagni

**Affiliations:** 1 COVID-19 National Epidemiology Cell, UK Health Security Agency, London, UK; 2 COVID-19 International Cell, UK Health Security Agency, London, UK; 3 COVID-19 National Guidance Cell, UK Health Security Agency, London, UK

**Keywords:** COVID-19, SARS-CoV-2, Surveillance, Quarantine, Isolation, Inequalities

## Abstract

**Background:**

From 12th March 2020, individuals in England were advised to quarantine in their home if a household member tested positive for SARS-CoV-2. A mandatory isolation period of 10 days was introduced on 28th September 2020 and applied to all individuals with COVID-19. We assessed the frequency, timing, and characteristics of recovered COVID-19 cases requiring subsequent quarantine episodes due to household re-exposure.

**Methods:**

In this case cohort study, all laboratory-confirmed COVID-19 cases notified in England (29th June to 28th December 2020) were analysed to identify consecutive household case(s). Multivariable logistic regression was used to determine associations between case characteristics and need to quarantine following recent infection (within 28 days of diagnosis).

**Results:**

Among 1,651,550 cases resident in private dwellings and Houses of Multiple Occupancy (HMOs), 744,548 (45.1%) were the only case in their home and 56,179 (3.4%) were succeeded by further household cases diagnosed within 11–28 days of their diagnosis. Of 1,641,412 cases arising in private homes, the likelihood of further household cases was highest for Bangladeshi (aOR = 2.20, 95% CI = 2.10–2.31) and Pakistani (aOR = 2.15, 95% CI = 2.08–2.22) individuals compared to White British, as well as among young people (17-24y vs. 25-64y; aOR = 1.19, 95% CI = 1.16–1.22), men (vs. women; aOR = 1.06, 95% CI = 1.04–1.08), London residents (vs. Yorkshire and Humber; aOR = 1.57, 95% CI = 1.52–1.63) and areas of high deprivation (IMD 1 vs. 10; aOR = 1.13, 95% CI = 1.09–1.19).

**Conclusion:**

Policies requiring quarantine on re-exposure differentially impact some of the most disadvantaged populations. Quarantine exemption for recently recovered individuals could mitigate the socioeconomic impact of responses to COVID-19 or similar infectious disease outbreaks.

## Introduction

On 12th March 2020, the UK government published quarantine guidance for individuals exposed to SARS-CoV-2 [1]. Quarantine refers to the separation of presently healthy contacts of infected individuals, whereas isolation is the separation of infected individuals, otherwise referred to as cases [2]. Close contacts, including all household members, were subject to quarantine to minimise the risk of onwards viral transmission. The self-isolation (cases) and quarantine (contacts) periods in the UK were changed from 7 to 14 days following the cases’ symptom onset or positive specimen date to 10 days in July and December 2020, respectively [1–3]. This policy applied to the entire population, including those with recent history of COVID-19, and remained in place until 16th August 2021 [4]. By this point, over 40 million UK residents had received at least two doses of a COVID-19 vaccine [5]. With vaccination shown to substantially reduce the risk of transmission [6], infection and severe outcomes [7] with SARS-CoV-2, quarantine was no longer deemed necessary for double vaccinated contacts. Select individuals, such as those aged under 18 years and six months, were also exempted from quarantine from August 2021 [4].

Studies have demonstrated that transmission of SARS-CoV-2 most commonly occurs within a residential environment and between known contacts [8, 9]. The requirement for a recently recovered individual to quarantine would therefore be most commonly triggered by the occurrence of secondary household cases. Given the likely short-term natural immunity acquired from infection [10], this quarantine period could be considered unnecessary, although concern would remain regarding potential re-exposure to a different strain from household members. Genetic discordance between SARS-CoV-2 infections among relatives and friends is uncommon if acquired within days of each other [11], thus questioning the need for the policy. Germany [12], Norway [13] and Cyprus [14] were among several countries to adopt quarantine exemptions on the basis of a “recovered” status, typically for individuals recovered from COVID-19 in the last six months. The emergence of novel COVID-19 variants will necessitate a review of such policies to assess if exemption on the basis of natural infection alone is appropriate; 9.5% of Omicron cases in the UK were detected among individuals with previous infection (≥ 90 days prior) as of 19 December 2021 compared to < 1.0% prior to the Omicron epidemic [15, 16].

Although isolation of an individual infected with SARS-CoV-2 and quarantine of their close contacts is essential to curtailing further transmission, domestic confinement can impact on the financial and broader wellbeing of the individual and society. A survey of 6,041 people in Canada identified that individuals who had to self-isolate or quarantine were significantly more likely to present with moderate to high stress, anxiety, and depressive symptomology compared to those who did not [17]. Moreover, 42.7% of 150 respondents living in London reported that they did not adhere to quarantine guidance, commonly because they required food provisions and lacked community support [2]. For some, the effects of social isolation may consequently be of greater concern than the prospect of transmitting SARS-CoV-2 onwards.

A 90-day period is widely used as the cut-off to define re-infection with SARS-CoV-2 on the basis of an assumed short-term period of immunity following initial infection [10]. However, while rare, re-infection has been observed within a period as short as three weeks [18]. Assuming a conservative natural immunity window of 28 days following infection with SARS-CoV-2, the number of avoidable instances of quarantine were a 28-day exemption policy to be retrospectively applied becomes measurable.

## Methods

### Study aim and design

In this case cohort study, we quantify the number of individuals diagnosed with COVID-19 who were succeeded beyond their own isolation period by further case(s) within the same household within 28 days of their diagnosis and characterise such individuals. In doing so, we assess the differential impact of quarantine policy.

### Participants and data sources

Data on laboratory-confirmed COVID-19 cases in England notified to Public Health England, as per statutory obligation, through national surveillance infrastructure for the period 29th June to 28th December 2020 were extracted [19]. Residential address data as reported at the time of testing, or National Health Service (NHS) summary care records if unavailable, were matched to Ordnance Survey reference databases to identify property type [20, 21]. Analyses were restricted to cases resident in private dwellings (Basic Land and Property Unit codes: RD, RD01-04, RD06-08, RD10, RH01-03, PP) and registered Houses of Multiple Occupancy (HMOs) (RH, RH01-03).

### Outcome variables

Singular cases were those where no further cases were identified at the same Unique Property Reference Number (UPRN), a unique identifier for each property in the UK [20], at the time of data extraction, 24th June 2021. Intervals between consecutive household cases were determined based on earliest positive specimen dates for each case. Individuals who had completed their isolation period following infection with SARS-CoV-2 were required to quarantine if they were re-exposed to a subsequent household case > 10 days after their own earliest positive specimen date.

### Explanatory variables

Associations between age, sex, ethnicity, region (Public Health England centre) and Index of Multiple Deprivation (IMD) decile with quarantine were assessed as these have been previously demonstrated to influence risk of infection and severe outcomes due to COVID-19 [22]. Further, as the aim of this study was to assess the differential impact of quarantine policy, the inclusion of these variables in statistical analyses enabled us to measure social inequities associated with quarantine. Age and sex were considered *a priori* confounders. Time period and housing type were also assessed as explanatory variables due to *a priori* hypotheses that likelihood of quarantine would vary according to the community incidence of COVID-19, circulating SARS-CoV-2 variants and the home environment, such as household composition and the ability to social distance.

### Statistical analysis

Univariable and multivariable logistic regression were used to model odds of further need to quarantine within 11–28 days, a period in which re-exposure would potentially be to the same SARS-CoV-2 strain, with likelihood ratio test used to assess significance of explanatory variables and goodness of fit. Variables associated with quarantine in univariable analyses were added to the multivariable model using a forward selection strategy and retained if model fit improved. Analyses were stratified by property type (private residential dwelling and HMO) to allow for potential differences in household composition; HMOs are typically rented by unrelated individuals, whereas those inhabiting private residential dwellings are more likely to be from the same family [23]. Analyses were restricted to cases occurring from 29th June to 28th December 2020, a period where community testing was widely available, the second epidemic wave was underway and the COVID-19 vaccination programme had yet to be rolled out. Time period was segmented according to the different phases of national restrictions [24]. Age was categorised according to life-course events, as previously described [25]. Ethnicity was categorised into White, Asian, Black, Mixed and Other for cases arising in HMOs; it was not possible to break down in smaller ethnicity categories due to small sample size at greater granularity. Relative deprivation was assessed using deciles of the IMD linked to residential lower super output area, preferentially by use of postcode data supplied by individuals at the time of SARS-CoV-2 testing [20]. Reference groups were chosen on the basis of largest sample size, apart from sex and IMD where male and the least deprived decile were selected for ease of interpretation. The 17–24 years age group was retained as the reference to maintain consistency across analyses stratified by property type.

## Results

**Fig. 1:**
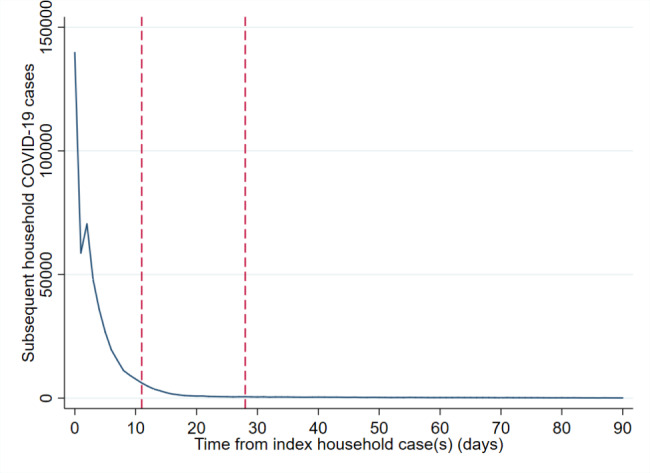
Time (days) until consecutive COVID-19 case(s) from index case(s) in residential dwellings, June-December 2020, England. (Legend: Exponential decline in consecutive household COVID-19 cases with time from index case, excluding a temporary increase on day two. Period of quarantine (11–28 days post COVID-19 diagnosis) assessed for index case indicated by dashed red lines on x-axis)

Of 1,651,550 cases that could be mapped to a known household address in England from 29th June to 28th December 2020, 1,641,412 (99.4%) occurred within a private residential dwelling and 10,138 (0.6%) within a HMO (see Table 1). Singular cases were common within residential dwellings (45.0%; 739,002), while some households had two (29.6%; 485,186), three (14.3%; 234,331), four (7.3%; 119,847) or five to 16 (3.8%; 63,046) cumulative cases by the time of data extraction. Where multiple cases at the same residence were identified, these most commonly occurred within 0–2 (329,007) or 3–10 (200,733) days of each other, with a further 55,726 arising within 11–28 days of each other, thus comprising the group theoretically eligible for quarantine after completing their own isolation period (see Fig. 1).

**Table 1:** Number and time (days) to consecutive COVID-19 case(s) within a household, June-December 2020, England

Total no. household cases^a^	Time (days) to subsequent household COVID-19 case						No. cases (%)
	**0–2**	**3–10**	**11–28**	**29–60**	**61–90**		
**Private residential dwelling**							
1	-	-	-	-	-		739,002 (45.0%)
2	129,033	81,252	20,287	10,120	7,762		485,186 (29.6%)
3	98,101	59,659	17,050	6,909	5,078		234,331 (14.3%)
4	63,149	36,853	10,385	3,468	2,615		119,847 (7.3%)
5	24,391	14,215	4,584	1,414	1,080		41,105 (2.5%)
6	8,729	5,232	1,972	653	423		13,690 (0.8%)
7	3,407	2,079	807	328	205		5,069 (0.3%)
8	1,324	807	350	171	72		1,896 (0.1%)
9	498	328	154	67	43		729 (0.04%)
10	234	219	110	74	22		379 (0.02%)
11	62	37	3	0	1		77 (0.01%)
12	18	11	2	10	2		24 (< 0.01%)
13	30	16	19	1	0		38 (< 0.01%)
14	12	10	0	0	0		14 (< 0.01%)
15	11	7	0	1	0		14 (< 0.01%)
16	8	8	3	11	1		11 (< 0.01%)
Total	329,007	200,733	55,726	23,227	17,304		1,641,412
**Houses of multiple occupancy**							
1	-	-	-	-	-		5,546 (54.7%)
2	479	278	159	130	89		2,308 (22.8%)
3	381	255	120	98	60		1,146 (11.3%)
4	255	149	77	50	23		553 (5.5%)
5	158	93	34	29	30		298 (2.9%)
6	91	63	31	16	5		163 (1.6%)
7	43	28	24	18	19		80 (0.8%)
8	11	8	2	1	1		16 (0.2%)
9	17	11	5	9	1		25 (0.2%)
10	0	0	1	2	2		3 (0.03%)
Total	1,435	885	453	353	230		10,138

^a^The total number of laboratory-confirmed COVID-19 household cases was based on data to 24th June 2021, whereas the number of re-exposed cases was restricted to cases diagnosed (by earliest positive specimen date) between 29th June and 28th December 2020

**Fig. 2:**
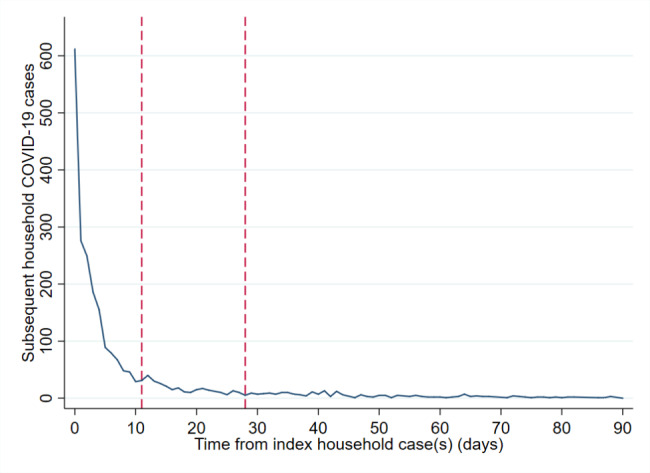
Time (days) until consecutive COVID-19 case(s) from index case(s) in HMOs^a^, June-December 2020, England. (^a^Houses of multiple occupancy. Legend: Exponential decline in consecutive household COVID-19 cases with time from index case. Period of quarantine (11–28 days post COVID-19 diagnosis) assessed for index case indicated by dashed red lines on x-axis)

Among cases living in HMOs most (5,546/10,138; 54.7%) were singular, with two cases detected in 2,308 (22.8%) households, three within 1,146 (11.3%), four within 553 (5.5%) and five to 10 within 585 (5.8%) households by the time of data extraction. Similarly to private residential dwellings, the majority of consecutive cases within HMOs occurred within 0–2 (1435) or 3–10 (885) days of each other, with 453, the quarantine group, arising within 11–28 days (see Fig. 2).

### Private residential dwellings

**Fig. 3:**
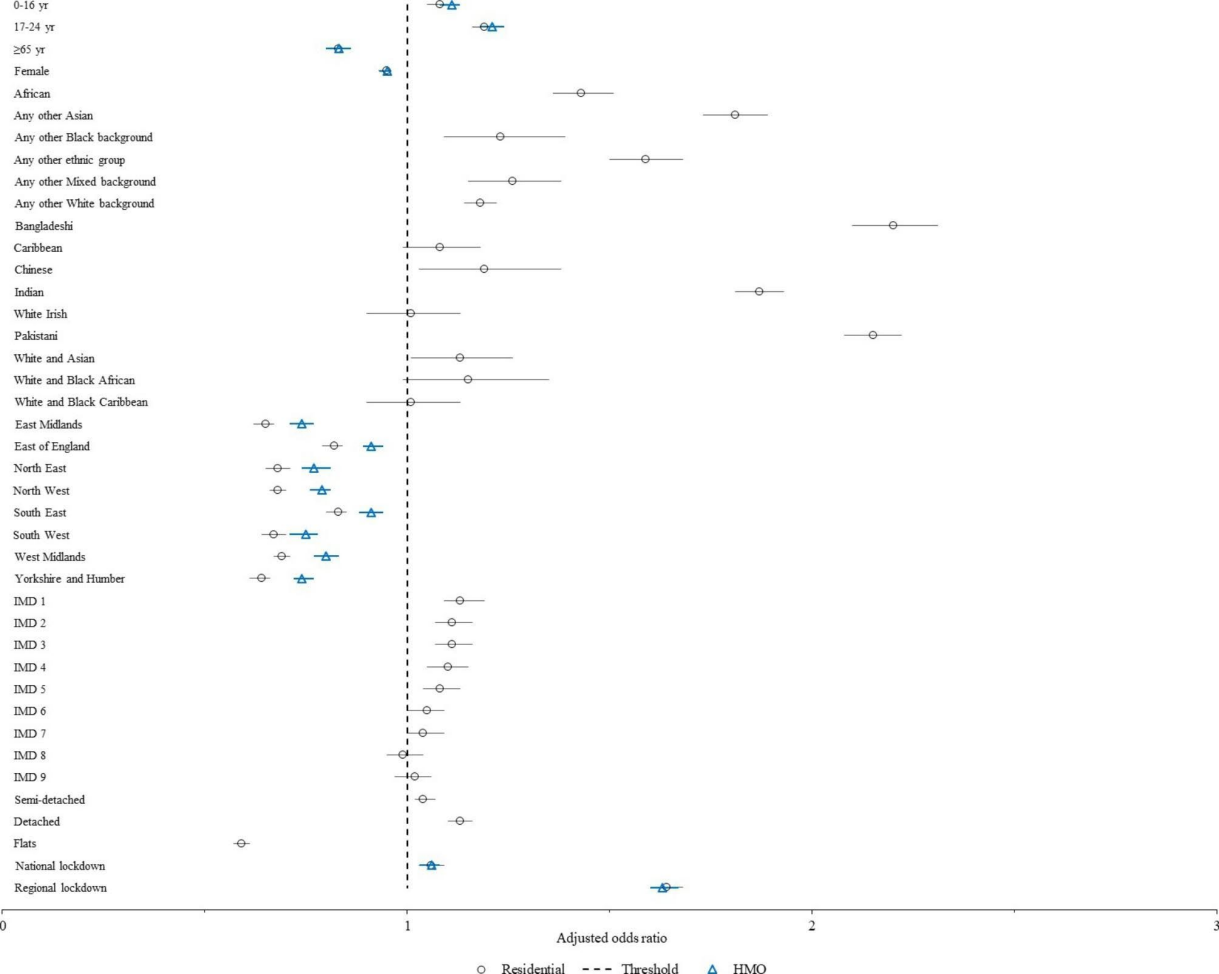
Adjusted odds ratio for quarantine by selected variables and property type, June-December 2020, England. (Legend: Adjusted odds ratios (aOR) with 95% confidence intervals for quarantine within 11–28 days of COVID-19 diagnosis for cases arising in private residential dwellings (black circle) and Houses of Multiple Occupancy (HMO) (blue triangle). Dashed vertical line indicates threshold for aORs greater than 1.0. Reference groups were as follows: 25–64 years, male, White British, London, Index of Multiple Deprivation (IMD) 10, terraced housing, time period of eased restrictions (29 June-4 November 2020). Adjusted odds ratios not presented by ethnicity (due to different strata) and IMD (as not included in final multivariable model) for HMOs)

The univariable and multivariable results were generally similar, as shown in Table 2. Cases aged 0–16 (adjusted odds ratio, aOR = 1.08, 95% CI = 1.05–1.11) and 17–24 (aOR = 1.19, 95% CI = 1.16–1.22) years old were more likely to be followed by further household cases within 11–28 days compared to those aged 25–64, while cases aged over 65 were least likely (aOR = 0.83, 95% CI = 0.80–0.86) (see Table 2; Fig. 3). Women were slightly less likely than men to have subsequent household cases within 11–28 days (aOR = 0.95, 95% CI = 0.93–0.96). Marked differences were seen according to cases’ ethnicity (compared to White British cases), ranking from highest to lowest as follows: Bangladeshi (aOR = 2.20, 95% CI = 2.10–2.31), Pakistani (aOR = 2.15, 95% CI = 2.08–2.22), Indian (aOR = 1.87, 95% CI = 1.81–1.93), other Asian background (aOR = 1.81, 95% CI = 1.73–1.89), any Other ethnic group (aOR = 1.59, 95% CI = 1.50–1.68), African (aOR = 1.43, 95% CI = 1.36–1.51), any other Mixed background (aOR = 1.26, 95 CI = 1.15–1.38), any other Black (aOR = 1.23, 95% CI = 1.09–1.39), Chinese (aOR = 1.19. 95% CI = 1.03–1.38), any other White background (aOR = 1.18, 95% CI = 1.14–1.22), and White and Asian (aOR = 1.13, 95% CI = 1.01–1.26) ethnicity.

**Table 2:** Characteristics of quarantined, recovered COVID-19 cases due to household re-exposure within 11–28 days, June-December 2020, England

	Total no. cases	No. quarantine cases			Quarantine (%)	OR (95% confidence interval)	aOR (95% confidence interval)	*p*
**Private residential dwelling**								
**Age (years)**								
0–16	199,912	7,840		3.9		1.18 (1.15–1.21)	1.08 (1.05–1.11)	< 0.001
17–24	240,659	9,025		3.8		1.13 (1.10–1.15)	1.19 (1.16–1.22)	< 0.001
25–64	1,041,239	34,773		3.3		1.00 (reference)	1.00 (reference)	-
≥ 65	158,922	4,049		2.5		0.76 (0.73–0.78)	0.83 (0.80–0.86)	< 0.001
**Sex**								
Male	758,122	26,589		3.5		1.00 (reference)	1.00 (reference)	-
Female	878,161	28,908		3.3		0.94 (0.92–0.95)	0.95 (0.93–0.96)	< 0.001
**Ethnicity**								
White British	1,108,042	30,663		2.8		1.00 (reference)	1.00 (reference)	-
African	38,124	1,631		4.3		1.57 (1.49–1.65)	1.43 (1.36–1.51)	< 0.001
Any other Asian background	41,591	2,309		5.6		1.98 (1.98–2.16)	1.81 (1.73–1.89)	< 0.001
Any other Black background	7,156	268		3.7		1.37 (1.21–1.55)	1.23 (1.09–1.39)	0.001
Any other ethnic group	29,875	1,433		4.8		1.77 (1.68–1.87)	1.59 (1.50–1.68)	< 0.001
Any other Mixed background	13,620	531		3.9		1.43 (1.31–1.56)	1.26 (1.15–1.38)	< 0.001
Any other White background	105,755	3,734		3.5		1.29 (1.24–1.33)	1.18 (1.14–1.22)	< 0.001
Bangladeshi	31,410	2,110		6.7		2.53 (2.42–2.65)	2.20 (2.10–2.31)	< 0.001
Caribbean	16,844	578		3.4		1.25 (1.15–1.36)	1.08 (0.99–1.18)	0.07
Chinese	5,195	190		3.7		1.33 (1.15–1.54)	1.19 (1.03–1.38)	0.02
Indian	80,372	4,468		5.6		2.07 (2.00–2.14)	1.87 (1.81–1.93)	< 0.001
White Irish	10,103	303		3.0		1.09 (0.97–1.22)	1.01 (0.90–1.13)	0.86
Pakistani	89,995	5,174		5.7		2.14 (2.08–2.21)	2.15 (2.08–2.22)	< 0.001
White and Asian	8,975	316		3.5		1.28 (1.15–1.44)	1.13 (1.01–1.26)	0.04
White and Black African	4,637	167		3.6		1.31 (1.12–1.53)	1.15 (0.99–1.35)	0.08
White and Black Caribbean	10,661	325		3.0		1.10 (0.99–1.23)	1.01 (0.90–1.13)	0.91
**Public Health England centre**								
London	305,713	14,837		4.9		1.00 (reference)	1.00 (reference)	-
East Midlands	138,618	3,804		2.7		0.55 (0.53–0.57)	0.65 (0.62–0.67)	< 0.001
East of England	161,192	6,199		3.8		0.78 (0.76–0.81)	0.82 (0.79–0.84)	< 0.001
North East	92,053	2,345		2.5		0.51 (0.49–0.54)	0.68 (0.65–0.71)	< 0.001
North West	278,947	7,661		2.7		0.55 (0.54–0.57)	0.68 (0.66–0.70)	< 0.001
South East	208,658	7,821		3.7		0.76 (0.74–0.79)	0.83 (0.80–0.85)	< 0.001
South West	84,180	2,238		2.7		0.54 (0.51–0.56)	0.67 (0.64–0.70)	< 0.001
West Midlands	176,249	5,665		3.2		0.65 (0.63–0.67)	0.69 (0.67–0.71)	< 0.001
Yorkshire and Humber	194,946	5,130		2.6		0.53 (0.51–0.55)	0.64 (0.61–0.66)	< 0.001
**Index of Multiple Deprivation decile**								
1 (most deprived)	201,088	6,495		3.2		1.01 (0.97–1.05)	1.13 (1.09–1.19)	< 0.001
2	196,151	6,978		3.6		1.12 (1.08–1.16)	1.11 (1.07–1.16)	< 0.001
3	189,439	7,012		3.7		1.17 (1.12–1.21)	1.11 (1.07–1.16)	< 0.001
4	172,063	6,228		3.6		1.14 (1.09–1.19)	1.10 (1.05–1.15)	< 0.001
5	161,895	5,702		3.5		1.11 (1.06–1.15)	1.08 (1.04–1.13)	< 0.001
6	151,579	5,112		3.4		1.06 (1.02–1.10)	1.05 (1.00–1.09)	0.04
7	148,944	4,898		3.3		1.03 (0.99–1.08)	1.04 (1.00–1.09)	0.05
8	148,720	4,617		3.1		0.97 (0.93–1.01)	0.99 (0.95–1.04)	0.72
9	142,870	4,597		3.2		1.00 (0.97–1.05)	1.02 (0.97–1.06)	0.44
10 (least deprived)	127,695	4,073		3.2		1.00 (reference)	1.00 (reference)	-
**Housing type**								
Terraced	545,497	20,157		3.7		1.00 (reference)	1.00 (reference)	-
Semi-detached	510,911	17,119		3.4		0.90 (0.88–0.92)	1.04 (1.02–1.07)	< 0.001
Detached	314,541	10,649		3.4		0.91 (0.89–0.94)	1.13 (1.10–1.16)	< 0.001
Flats	223,903	5,876		2.6		0.70 (0.68–0.72)	0.59 (0.57–0.61)	< 0.001
**Time period**								
Eased restrictions (29th June to 4th November 2020)	610,426	15,965		2.6		1.00 (reference)	1.00 (reference)	-
National lockdown (5th November to 2nd December 2020)	424,246	11,917		2.8		1.08 (1.05–1.10)	1.06 (1.03–1.09)	< 0.001
Regional lockdowns (3rd to 28th December 2020)	606,740	27,844		4.6		1.79 (1.76–1.83)	1.64 (1.61–1.68)	< 0.001
**Houses of multiple occupancy**								
**Age (years)**								
0–16	328	16		4.9		1.03 (0.61–1.74)	1.11 (1.08–1.13)	< 0.001
17–24	5,955	259		4.3		0.91 (0.75–1.11)	1.21 (1.18–1.24)	< 0.001
25–64	3,685	175		4.7		1.00 (reference)	1.00 (reference)	-
≥ 65	165	3		1.8		0.37 (0.12–1.18)	0.83 (0.80–0.86)	< 0.001
**Sex**								
Male	4,782	207		4.3		1.00 (reference)	1.00 (reference)	-
Female	5,312	244		4.6		1.06 (0.88–1.29)	0.95 (0.93–0.96)	< 0.001
**Ethnicity**								
White British	6,901	289		4.2		1.00 (reference)	1.00 (reference)	-
Asian or Asian British	1,360	67		4.9		1.19 (0.90–1.56)	1.97 (1.93–2.01)	< 0.001
Black or Black British	558	29		5.2		1.25 (0.85–1.86)	1.22 (1.17–1.28)	< 0.001
Mixed or Mixed British	373	10		2.7		0.63 (0.33–1.19)	1.09 (1.03–1.15)	0.004
Any Other ethnicity	277	18		6.5		1.59 (0.97–2.60)	1.49 (1.41–1.58)	< 0.001
**Public Health England centre**								
London	1,760	99		5.6		1.00 (reference)	1.00 (reference)	-
East Midlands	938	37		3.9		0.69 (0.47–1.01)	0.74 (0.71–0.77)	< 0.001
East of England	643	43		6.7		1.20 (0.83–1.74)	0.91 (0.89–0.94)	< 0.001
North East	96	1		1.0		0.18 (0.02–1.28)	0.77 (0.74–0.81)	< 0.001
North West	1,601	64		4.0		0.70 (0.51–0.96)	0.79 (0.76–0.81)	< 0.001
South East	1,348	53		3.9		0.69 (0.49–0.97)	0.91 (0.88–0.94)	< 0.001
South West	1,174	58		4.9		0.87 (0.63–1.22)	0.75 (0.71–0.78)	< 0.001
West Midlands	843	37		4.4		0.77 (0.52–1.13)	0.80 (0.77–0.83)	< 0.001
Yorkshire and Humber	1,717	61		3.6		0.62 (0.45–0.86)	0.74 (0.72–0.77)	< 0.001
**Index of Multiple Deprivation decile**								
1 (most deprived)	708	25		3.5		0.79 (0.42–1.51)	-	-
2	926	55		5.9		1.37 (0.77–2.42)	-	-
3	1,718	79		4.6		1.05 (0.60–1.81)	-	-
4	1,407	72		5.1		1.17 (0.67–2.04)	-	-
5	1,363	52		3.8		0.86 (0.49–1.53)	-	-
6	1,360	54		4.0		0.90 (0.51–1.59)	-	-
7	905	38		4.2		0.95 (0.52–1.73)	-	-
8	810	36		4.4		1.01 (0.55–1.84)	-	-
9	547	25		4.6		1.04 (0.55–1.97)	-	-
10 (least deprived)	363	16		4.4		1.00 (reference)	-	-
**Time period**								
Eased restrictions (29th June to 4th November 2020)	5,453	222		4.1		1.00 (reference)	1.00 (reference)	-
National lockdown (5th November to 2 December 2020)	2,212	86		3.9		0.95 (0.74–1.23)	1.06 (1.03–1.08)	< 0.001
Regional lockdowns (3 December to 28 December 2020)	2,473	145		5.9		1.47 (1.18–1.82)	1.63 (1.60–1.67)	< 0.001

Legend: Univariable and multivariable analysis of variables associated with quarantine within 11–28 days of COVID-19 diagnosis. Variables associated with quarantine in univariable analyses were added to the multivariable model using a forward selection strategy and retained if model fit improved. T-test p-values show associations between variable coefficients and quarantine in the final multivariable model. Likelihood ratio test statistics not shown

Compared to London, all other regions observed lower likelihood of subsequent household cases within 11–28 days, with Yorkshire and Humber lowest of all (aOR = 0.64, 95% CI = 0.61–0.66). An increasing gradient in likelihood of subsequent household cases within 11–28 days was seen according to increasing levels of socioeconomic deprivation (IMD 1 vs. 10; aOR = 1.13, 95% CI = 1.09–1.19). Compared to cases living in terraced houses, those living in semi-detached (aOR: 1.04, 95% CI: 1.02–1.07) and detached (aOR = 1.13, 95% CI = 1.10–1.16) housing had higher likelihood of further cases within 11–28 days, whereas flats were least likely to give rise to further cases (aOR = 0.59, 95% CI = 0.57–0.61).

Odds of quarantine increased during periods of lockdown compared to eased restrictions, when the national incidence of COVID-19 was lower (3rd -28th December 2020 vs. 29th June-4th November 2020, aOR: 1.64, 95% CI = 1.61–1.68).

### HMO

A similar age pattern was seen as for cases aged 0–16 (aOR = 1.11, 95% CI = 1.08–1.13) and 17–24 (aOR = 1.21, 95% CI = 1.18–1.24) having higher odds of subsequent cases within 11–28 days compared to those aged 25–64, while those aged over 65 had reduced odds (aOR = 0.83, 95% CI = 0.80–0.86) (see Table 2). Women were again less likely to be succeeded by further cases within 11–28 days than men (aOR = 0.95, 95% CI = 0.93–0.96), and cases of Asian or Asian British (aOR = 1.97, 95% CI = 1.93–2.01), Black or Black British (aOR = 1.22, 95% CI = 1.17–1.28), Mixed or Mixed British (aOR = 1.09, 95% CI = 1.03–1.15) and any Other ethnicity (aOR = 1.49, 95% CI = 1.41–1.58) had increased odds of quarantine within 11–28 days compared to their White British counterparts. Regional and temporal patterns were very similar to those for private dwellings, with cases in London and those arising later in 2020 most likely to be succeeded by further household cases within 11–28 days.

## Discussion

This case cohort study measured the frequency, timing, and characteristics of recovered COVID-19 cases who would have had subsequent quarantine episodes due to household re-exposure. We identified 56,179 laboratory-confirmed cases of COVID-19 diagnosed between 29th June and 28th December 2020 who were succeeded by a further case(s) at the same residence within 11–28 days of their earliest positive specimen date, rendering one in 29 household cases subject to quarantine almost immediately after their own isolation period at the time.

After adjusting for potential confounding, there was evidence of an association between age, sex, ethnicity, region, IMD, housing type and time period with odds of further household cases arising within a private residential dwelling within 11–28 days of each case’s diagnosis. Similarly, age, sex, ethnicity, region, and time period were associated with likelihood of subsequent household cases in HMOs within 11–28 days. Across private residential dwellings and HMOs, young recovered cases were 19% and 21% more likely to be subject to quarantine within 11–28 days compared to adults aged 25–64, respectively. A disproportionate number of individuals of Asian or Asian British ethnicity were succeeded by a consecutive household case, particularly those of Bangladeshi, Pakistani, and Indian ethnicity, who had a two-fold increased likelihood of quarantine within 11–28 days compared to White British individuals. More moderate increased odds of quarantine were observed among those of Black British, Mixed and Other ethnicity, as well as among men. Regionally, London was the most impacted area and, among cases arising in residential dwellings, those living in the most deprived areas had up to 13% increased likelihood of having to quarantine, as did those living in detached housing.

The substantial divergence in quarantine requirement by personal characteristics sheds light on earlier reports of unequal health outcomes relating to COVID-19. In Public Health England’s review of disparities in risks and outcomes of COVID-19, individuals of Black or Bangladeshi ethnicity were found to be over twice as likely to be diagnosed or die with COVID-19 compared to their White counterparts, respectively [22]. Higher rates of over-crowded housing, obesity and deprivation among ethnic minorities have been posited as possible explanations of these findings [26, 27]. Approximately 60% of retirement-age households contained only one person in a study of 19,425 UK households, a likely explanation for why we found individuals aged 65 and over to have the lowest odds of quarantine [28]. Living in a multi-generational household has been linked to a higher risk of infection with SARS-CoV-2, and consequently mortality [21, 29, 30]. As some ethnic minority populations are more likely to live in large, multi-generational households, particularly those of Bangladeshi, Pakistani or Indian ethnicity [27, 29], household composition, in addition to size, may also explain the differences observed across ethnic groups. Indeed, recovered cases living in London, where median household size and multigenerational housing is highest in England [25], had the greatest likelihood of being asked to quarantine in our study. We note that the English Housing Survey found that after adjusting for income, average household size was still larger among ethnic minorities compared to those of White ethnicity, indicating that multi-generational living is as much a personal and cultural decision as it is an economic one [27]. While larger household size increases risk of infection, and likelihood of quarantine, living with more people can protect against the detrimental psychosocial impacts of social isolation [31].

As well as household size, proximity within the household and relationship type, such as familial, were also likely to have influenced the possibility of quarantine; further household cases were less common in HMOs than private residential dwellings, but the temporal distribution of subsequent household cases in HMOs was longer than in residential dwellings. Cases who were able to effectively isolate may have avoided transmitting the infection to other household members, as previous studies have suggested [25], but were more likely to be re-exposed to SARS-CoV-2 at a later point. Increased odds of quarantine were also observed as the year progressed, supporting earlier findings of increased transmissibility of the Alpha variant, first detected in the UK in November 2020 and the predominant lineage by February 2021 [32, 33].

Our analysis was not without limitations. Although six months’ of national surveillance data comprising over 1.6 million individuals were included, our analysis was based on individuals who tested positive for COVID-19 in 2020. As such, household size could not be adjusted for as denominator data were not available. Our findings are specific to the time period studied with population-level immunity (natural or vaccine-induced) and prevailing variant characteristics each likely to affect secondary transmission patterns [6]. Individuals who were not willing or not able to test for COVID-19 would also be under-represented. Moreover, as national surveillance data were de-duplicated to be person-level, we could not identify possible repeat infections and explore the appropriateness of using 28 days as a window of post-infection protection.

## Conclusion

Differential morbidity, mortality [22], vaccine uptake [34] and, according to our findings, likelihood of quarantine due to short-term re-exposure to SARS-CoV-2, by age, sex, ethnicity, region, housing type and deprivation risk exacerbating existing health inequalities in England. Financial support was made available through the NHS Test and Trace Support Payment Scheme after 28th September 2020 for low-earning individuals obliged to isolate or quarantine and [35], for some, as early as 1st March 2020 through statutory sick pay or furlough [36, 37]. However, the cost of repeated social isolation was likely to have been disproportionately felt by some of society’s most disadvantaged. Studies have demonstrated how the detrimental impacts of self-isolation may be mitigated through the use of rapid antigen testing to gauge infectiousness and identify those eligible for early release, should self-isolation measures be reconsidered for resurgences of COVID-19 and similar infectious diseases [38, 39]. While vaccination remains the mainstay for protection against (re)infection with, transmission of, and severe disease due to SARS-CoV-2 [40], recent history of COVID-19 offers at least some protection in the short-term [10]. The comparatively low risk posed by individuals with very recent history of COVID-19 should therefore be considered alongside our assessment of differential impact of quarantine policies, as well as evolving understanding through emerging variants, in setting future strategies to control spread of SARS-CoV-2.

## Data Availability

The datasets used in our study are confidential records supplied to UK Health Security Agency under Regulation 3 of The Health Service (Control of Patient Information) Regulations 2020 and under Sect. 251 of the NHS Act 2006. In accordance with our duty of confidentiality, requests for access will be considered but subject to legal restrictions. Custom code using Stata v15.1 (StataCorp LP, College Station, TX, USA). The code for the current study is available from the corresponding author on reasonable request.

## Abbreviations

*aOR*: adjusted Odds Ratio.
*HMOs*: Houses of Multiple Occupancy.
*IMD*: Index of Multiple Deprivation.
*95% CI*: 95% Confidence Interval.
